# Perceptions and attitudes regarding delayed antibiotic prescription for respiratory tract infections: a qualitative study

**DOI:** 10.1186/s12875-023-02123-4

**Published:** 2023-10-04

**Authors:** Gemma Mas-Dalmau, Sandra Pequeño-Saco, Mariam de la Poza-Abad, Eulàlia Borrell-Thió, Marta Besa-Castellà, Maria Alsina-Casalduero, Lluís Cuixart-Costa, Mercedes Liroz-Navarro, Carlos Calderón-Gómez, Joel Martí, Irene Cruz-Gómez, Pablo Alonso-Coello

**Affiliations:** 1 Epidemiology and Public Health Department - Iberoamerican Cochrane Centre, Hospital de la Santa Creu i Sant Pau - Biomedical Research Institute Sant Pau (IIB Sant Pau), Barcelona, Spain; 2 Nursing Care Research Group, IIB Sant Pau, Barcelona, Spain; 3 Sub- Directorate General of Surveillance and Response to Public Health Emergencies, Public Health Agency of Catalonia, Generalitat of Catalonia, Barcelona, Spain; 4 Doctor Carles Ribas Primary Care Centre, Barcelona, Spain; 5 Sant Roc Primary Care Centre, Badalona, Spain; 6 Florida Sud Primary Care Centre, Hospitalet de Llobregat, Barcelona, Spain; 7 Llefià Primary Care Centre, Badalona, Spain; 8 PADES (Domestic Care Program, Support Teams) Mutuam Group, Barcelona, Spain; 9 Roger de Flor Primary Care Centre, Barcelona, Spain; 10 Alza Primary Care Centre, San Sebastián, Spain; 11 Centre d’Estudis Sociològics sobre la Vida Quotidiana i el Treball (QUIT), Institut d’Estudis del Treball (IET), Universitat Autònoma de Barcelona, Bellaterra, Spain; 12 CIBER Epidemiology and Public Health, Instituto de Salud Carlos III (CIBERESP), Madrid, Spain

**Keywords:** Qualitative research, Primary care, Professionals, Antibiotics, Delayed antibiotic prescription

## Abstract

**Background:**

Antibiotics are overprescribed for respiratory tract infections (RTIs). However, the decision to prescribe is often complex. Delayed antibiotic prescription (DAP), a strategy designed to promote more rational antibiotic use, is still not widely used. The aim of this study was to explore perceptions and attitudes in primary care professionals, regarding antibiotic use and different DAP strategies for uncomplicated RTIs.

**Methods:**

We conducted a qualitative study, using an inductive thematic approach to generate themes, based on focus group discussions and semi-structured interviews with professionals, recruited from 6 primary care centres (Barcelona metropolitan area, Spain).

**Results:**

26 professionals (25 family physicians and one nurse) were included in four focus group discussions and three semi-structured interviews. Participants commented that RTIs were a main reason for consultation, motivated often by patient anxiety and fear of possible complications, and this was associated with the patients’ poor health-related education. Acknowledging inappropriate antibiotic use in the health system, participants attributed this, mainly to defensive medicine strategies. DAP was used when in doubt about the aetiology, and considering factors related to patient-physician interactions. The main perceived advantage of DAP was that it could reduce the need for additional visits, while the main disadvantage was uncertainty regarding proper use by the patient.

**Conclusions:**

DAP was used by participants in cases of doubt, in specific situations, and for specific patient profiles. Weak points were detected in our primary care system and its users that affect the proper use of both antibiotics and DAP, namely, time pressure on professionals, poor patient health-related education, and the lack of a patient-physician relationship in some scenarios.

**Supplementary Information:**

The online version contains supplementary material available at 10.1186/s12875-023-02123-4.

## Introduction

Respiratory tract infections (RTIs), the most frequent infections encountered in primary care [1], are mostly self-limiting and are caused by viruses. While antibiotics may slightly modify course [2, 3], they tend to be overprescribed [4, 5]. Overuse of antibiotics is closely related with antimicrobial resistance [6–8], by now a major global public health challenge [9] that entails an increased risk of adverse effects for patients [10] and increased beliefs of the need to consult for similar episodes [11, 12]. In a context of optimal use of antibiotics, the decision to prescribe is complex, as, in some cases, symptoms are unclear; furthermore, the decision also depends on factors related to patient-physician interactions [4, 13], such as pressures from the patient [14, 15] and the patient-physician relationship [15].

One approach to reducing inappropriate antibiotic use for RTIs is delayed antibiotic prescription (DAP) [16], a strategy designed to promote a more rational antibiotic use in situations of uncertainty, regarding the need of immediate antibiotic prescription (IAP). DAP consists of the patient only using the antibiotic prescription if the RTI has not improved or has worsened, some days after consultation. A recent systematic review [17] comparing DAP, IAP, and no antibiotic prescription (NAP), reported that RTI symptom severity was similar for the 3 strategies, that symptom duration was slightly shorter for IAP versus DAP, and that re-visit and complication rates were lower and patient satisfaction was higher for DAP versus NAP.

DAP is still not widely deployed by professionals, as reported by several qualitative studies that have investigated views and experiences of DAP for RTIs among professionals in northern Europe [13, 18, 19], United States [18], Australia [20–22], and New Zealand [23]. While a study conducted by our group suggests that under 50% of primary care professionals in Spain use the DAP strategy [24], to our knowledge, no qualitative research evidence is available regarding this issue in Spain. Our objective was, therefore, to explore perceptions and attitudes of professionals regarding use of antibiotics and of different DAP strategies for noncomplicated RTIs.

## Methods

### Study design

Qualitative study using focus group discussions (FGDs) and semi-structured interviews [25–27], performed in primary care centres in the Barcelona metropolitan area (Spain).

### Participants and recruitment

Family physicians were recruited from six primary care centres, five of which had previously participated in the DAP-Trial [11]. This trial, conducted with adults with uncomplicated RTIs in a primary care setting, assessed the efficacy and safety of IAP versus NAP, and versus two DAP strategies: a delayed patient-led strategy (the patients receives the prescription, with instructions to only use it if the RTI worsens or fails to improve), and a delayed collection strategy (the patients collects their prescription from the primary care centre 3 days after the first visit if they consider they need it).

Sampling was purposive, with participants selected according to a strategy in which sample design was based on a theoretical construct [25, 27]. The criteria to define professional profiles that reflected possibly different discourses, were as follows: (a) the professional’s previous participation in the DAP-Trial (yes/no); and (b) the socioeconomic level of the professional’s primary care centre’s catchment population (medium-low/medium-high). Socioeconomic level was taken as a proxy for the education level of patients [28], as previous studies have shown that professionals do not consider DAP to be appropriate for less educated patients [21, 23]. For this reason, professionals were selected according to the deprivation index of the primary care centre’s catchment area [29]. The sample was demographically as heterogeneous as possible in terms of gender (women/men) and age (junior: <45 years/ senior: ≥45 years).

Candidate participants for this study were recruited by the DAP-Trial centre coordinators. A sample size of 24–36 participants was estimated as necessary (4 FGDs based on 6–9 participants); however, the final number of included participants was determined once data saturation was reached.

### Data collection

The study was conducted in two phases: first, focus group discussions, aimed at fostering interaction between participants [25–27]; and individual semi-structured interviews afterwards [25, 27]. In phase I, the participants completed a questionnaire about sociodemographic data and use of DAP strategy in their clinical practice.

#### Focus group discussions

FGDs were profiled according to sampling criteria as follows: FGD1, DAP-Trial participants and medium-low socioeconomic area; FGD2, DAP-Trial participants and medium-high socioeconomic area; FGD3, DAP-Trial non-participants and medium-low socioeconomic area; FGD4, DAP-Trial non-participants and medium-high socioeconomic area. In relation to FGD2, not enough family physicians were recruited. Thus, one nurse participating in the DAP-Trial was included. Even though her role with the antibiotics was different, we considered that her opinion could also be relevant because in many centres, the nurses carry out triage consultations. Similarly, their educational work and their experience in the trial was deemed relevant. A script was prepared for this study (**Appendix 1**) that was sufficiently flexible for participants to suggest new topics. FGDs, run with a moderator and an observer, were conducted in a meeting room in the coordinating centre (Hospital de la Santa Creu i Sant Pau (HSCSP) in Barcelona, Spain). All FGDs were digitally audiorecorded, and recordings were transcribed verbatim. Notes taken by the moderator and observer were also used, to check and complement the transcriptions’ data.

#### Individual semi-structured interviews

Semi-structured interviews were guided by a specifically designed script for this study (**Appendix 2**). They were carried out in order to further explore key issues that emerged in the FGDs. Interviews were conducted in the primary care centres where the participants worked with professionals drawn from the FGDs. The same researcher who moderated all FGDs also conducted the semi-structured interviews.

### Data analysis

The transcriptions were cross-checked against the digital recordings and inductive thematic analysis was performed as described by Braun and Clarke [30]. The analysis was conducted by 3 researchers. Two of them independently analysed the transcription of FGD1 and agreed a preliminary coding frame. The analysis of the other transcriptions was conducted by one researcher and a second researcher reviewed the coding. The discrepancies about emergent themes and codes were resolved by consensus between the researchers. We used ATLAS.ti (version 8) software for data coding and analysis. Quotations from the FGDs and interviews were translated from Catalan or Spanish to English. Investigator triangulation and search for negative cases were undertaken to improve rigour of the analysis [31].

## Results

We conducted 4 FGDs and 3 individual interviews, with a total of 26 participants, 25 physicians and 1 nurse, with a mean (SD) age of 46.81 (8.56) years, 13 (50%) worked in a primary care centre in a medium-low socioeconomic area and 12 (46.15%) previously participated in the DAP-Trial (Table 1).

**Table 1:** Sociodemographic characteristics of study participants (N = 26)

Participant characteristics	Frequency (%)
**Professional profile**	
Family physician	25 (96.15%)
Nurse	1 (3.85%)
**Gender**	
Woman	19 (73.08%)
Man	7 (26.92%)
**Age**	
Junior	13 (50%)
Senior	13 (50%)
**Socioeconomic level of the centre’s population**	
Medium-Low	13 (50%)
Medium-High	13 (50%)
**Participant in the DAP-Trial**	
Yes	12 (46.15%)
No	14 (53.85%)
**Use of DAP strategy** (even if only occasionally)	
Yes	19 (73.08%)
No	7 (26.92%)

The FGDs were conducted between September 2013 and June 2014. Mean duration was 90 min, except for FGD2, which lasted 60 min. Note that 2 physicians in FGD1 and 1 physician in FGD2 belonged in primary care centres with a different socioeconomic level from the rest of participants in their groups. The semi-structured interviews were conducted between October and December 2018 and lasted approximately 60 min.

We identified 4 main themes arising in the 4 FGDs and the 3 interviews: (1) Characteristics of RTI visits; (2) Expectations and adequacy of antibiotic treatment; (3) DAP, how and for whom; and (4) DAP-Trial and primary care research barriers. Example quotes are shown in Tables 2 and 3.

**Table 2:** Illustrative quotes:*Characteristics of RTI’s visits* and *Expectations and adequacy of antibiotic treatment*

*Major theme*	*Subtheme*	*Quotations*
***Characteristics of RTI’s visits***	*The concept of RTI*	*It is one of the main reasons for the consultations we get, and they involve many hours and many visits to address the reasons for consultation, most of which are trivial, and wouldn’t require them to come, but they all come…here.* (Professional(P)3, DAP-Trial participant, medium-high socioeconomic area) *(…) I mean apparently it does not seem to be a serious or very complicated disease most of the times, but in daily practice, it is quite demanding.* (P13, DAP-Trial non-participant, medium-low socioeconomic area) *And then they say to you “oh, well, the virus again—when you don’t know what I’ve got, I’ve always got a virus”. That has been said to me.* (P25, DAP-Trial non-participant, medium-high socioeconomic area)
	*RTI visits*	*Sometimes I don’t know, because they come so often with early symptoms, and one doesn’t know what’s going to happen after 24 hours, right? There are people who come, let’s say, in the ”prodromal stage” of the disease, right?, and you think “well, I don’t know”.* (P2, DAP-Trial participant, medium-low socioeconomic are)) *They are congested with an upper airway cold, but then “if it goes down to my chest”, things get very complicated. Well, I don’t know, sometimes they have a history of pneumonia or more serious problems, and then this…* (P13, DAP-Trial non-participant, medium-low socioeconomic area) *Colds, as you say, and gastroenteritis, these used to be resolved at home, and now people go to the doctor.* (P9, DAP-Trial participant, medium-high socioeconomic area) *Yes, I’d say we do secondary education, right?, and the potential complications. But at a primary prevention level, well, yes, more healthcare education should be conducted at the healthcare level as well as from mass media, other institutions, right?… I don’t know… in adult day care, at schools or… In order to improve self-care and knowing that with an — apparently unimportant— cold, people with no other illnesses or complications, they shouldn’t first go to the doctor or the healthcare centre.* (P4, DAP-Trial participant, medium-low socioeconomic area) … *since they are going to solve it for me, I don’t need to try to be more self-sufficient.* (P4, DAP-Trial participant, medium-low socioeconomic area)
***Expectations and adequacy of antibiotic treatment***	*Physician-indicated treatment*	*In a patient with an uncomplicated acute infection, if this patient has no risk factors and is not very old, then a minimal examination, and depending on the symptoms, then the treatment… at most, a symptomatic treatment with paracetamol and a mucolytic if they have a lot of mucus; or if they have sneezing and congestion symptoms, an antihistamine, and so on…* (P1, DAP-Trial participant, medium-high socioeconomic area) *Supposedly at least a viral presentation and the treatment…it should be with paracetamol*. (P13, DAP-Trial non-participant, medium-low socioeconomic area)
	*Inappropriate antibiotic use*	*Also with regard to the clinicians, there may have been a bit of defensive medicine, right?, In order to play it safe, we prescribe antibiotics so they won’t come back, or to satisfy the patient, or, I don’t know, this has been going on for a long time too.* (P14, DAP-Trial non-participant, medium-low socioeconomic area) *I believe that it is quite rational now, compared to 10 years ago. For instance, I believe that now we prescribe perhaps 10 times less antibiotics. In my opinion, I don’t know what the statistics say, but I think we prescribe antibiotics much less often now than 10 or 15 years ago.* (P19, DAP-Trial non-participant, medium-low socioeconomic area) (…) *And then, we often visit this type of patient profile in the unscheduled visits where not even the same doctor visits them. So the credibility of the professional here counts for a lot. For me, it’s much easier to work with my usual patients than when I visit with someone else.* (P23, DAP-Trial non-participant, medium-high socioeconomic area) *The mindset in England or Germany is not the same as here, where since I was a child I have had the feeling that they are used to taking antibiotics relatively often. It’s not their fault either, but also maybe there hasn’t been a good education. They come in a second time, this second visit you give it [the antibiotic] them so they won’t come back, I don’t know, sometimes we are all a little guilty.* (P20, DAP-Trial non-participant, medium-high socioeconomic area)
	*Patient self-medication*	*Many times they say, “no, I’m already taking paracetamol, aren’t I?“, then, —“then continue, very well”— “but I’m not cured” —“wait… wait a few days, and you’ll see, right?“* (P23, DAP-Trial non-participant, medium-high socioeconomic area) *A minority [has already started antibiotic treatment]. Pills left over from the last time, or from their grandmother.* (P11, DAP-Trial participant, medium-high socioeconomic area)

**Table 3:** Illustrative quotes: *DAP, how and for whom*; and *DAP-Trial and primary care research barriers*

Main theme	Subtheme	Quotations
***DAP, how and for whom***	*Context*	*Yes, because [the DAP] is something we use when it’s not entirely clear to us whether the presentation is going to resolve easily. When you have the slightest suspicion, a medical sixth sense that tells you, “hmm, this could get complicated”, or you’re not sure about a tonsillitis and you say, “well, look, it’s quite likely that, with so much pain, so much fever, in 48 hours, you’ll have an abscess or have terrible pus plaques.“ And you are not very sure. Basically, that’s it. An uncertainty that it may be something viral that will get complicated or that is already a bacterial infection.* (P10, DAP-Trial participant, medium-high socioeconomic area) *I have done it before the weekend. If they come in on a Monday, then you know it’s Monday, it’s fine because there is a lot of accessibility. I’ve done it more often on Thursdays, Fridays, thinking “where will they go on Saturday, Sunday?”. Before a public holiday or Easter holidays. When I’ve done it, I’ve done it more often in those situations.* (P22, DAP-Trial non-participant, medium-high socioeconomic area) *There are also times you use it as a tool not to prescribe immediately. In other words, you think they shouldn’t take it, the patient, you know they do not agree, so… and then you can use it in such a way, that a possibility, in the long run, maybe, is if they see that they are getting better, they won’t take it, and they don’t take it.* (P4, DAP-Trial participant, medium-low socioeconomic area) … *I believe it is more often in a situation of a quick unscheduled visit (…). Because you probably will not see this patient again, another professional will visit them instead, you lose follow-up of them. It’s ‘right here, right now’, another decision of the moment. If this happens with your patient, it’s easier to say “if you are not feeling well, come back in a few days and I will examine you again”.* (P4, DAP-Trial participant, medium-low socioeconomic area)
	*Patient profiles*	*That they really understand that they understand, or that one knows how to explain it to them, and they understand it (…)* (P13, DAP-Trial non-participant, medium-low socioeconomic area) *Those of us who have been working for a long time now, when we know the patient. Because, of course, when you’ve been working for a long time, you know if they are a compliant patient, if they are a multi-frequenter patient, if they are…… You know these things. If you think they are compliant and will do well, then that is also a criterion. If they are multi-frequenters, they’ll still come back after two days if they don’t get better, even with the DAP, that can also influence whether you do it or not. I mean, those are criteria that you can also consider.* (P9, DAP-Trial participant, medium-high socioeconomic area) *It is the profile of the people. I think that perhaps the population that could benefit most is the young population, who can understand it. But this population rarely comes to see us. And then, when you have to educate a patient with whom you aren’t too close, because the confidence your patients have in you is different. And then, we often visit with this type of patient profile in the unscheduled visits, where not even the same doctor visits them. So the credibility of the professional here counts a lot. For me, it is much easier to work with my usual patients than when I visit with someone else.* (P23, DAP-Trial non-participant, medium-high socioeconomic area) *In short, DAP is probably very suitable for patients who do not want antibiotics. This kind will wait 24 or 48 h. In other words, they are aware of not taking antibiotics. On the other hand, with those convinced of taking them, it doesn’t matter if you ask them to wait.* (P26, DAP-Trial non-participant, medium-high socioeconomic area) *The problem is that you still have a doubt, right?, with the patient who doesn’t agree, who’s not sure… or if one thinks or this person is a hypochondriac, that once I give them a DAP, they will accept it, and will surely go directly to buy an antibiotic. Because I don’t leave the prescriptions at the reception desk, I give them in person. And then you doubt, right?* (P7, DAP-Trial participant, medium-low socioeconomic area)
	*DAP advantages and disadvantages*	*Let’s see, advantages… You could say the number of visits, maybe, but I don’t care. In other words, if the patient is not feeling well, it’s fine that they come back and visit me again. This is the advantage I can think of.* (P15, DAP-Trial non-participant, medium-low socioeconomic area) *(…) I think they find this option safer for them, don’t they? [they think,] “OK, you now think it is not necessary, but you let me this second option in case I get worse…”* (P4, DAP-Trial participant, medium-low socioeconomic area) (…) *Anyway, I use it and I use it also for that reason (…) they are no longer in distress thinking “I feel terrible”, and it also gives them the chance to say, “well, maybe I don’t need it, the antibiotic, right?“ And therefore, well, I don’t know, it’s useful, it’s useful. And the patient, from what I see, leaves satisfied.* (P4, DAP-Trial participant, medium-low socioeconomic area) *(…) what’s most important to me: you give them a little independence and self-management of their own health. And actually the only thing you are doing differently is knowing that they must take the antibiotic if it happens to them or not, I mean, if you explain it to them, they are able to do it themselves. Not everyone, though.* (P3: DAP-Trial participant, medium-high socioeconomic area) *The problem is that you still have a doubt, right? With the patient who doesn’t agree, who’s not sure, or if one thinks that this person is a hypochondriac, that once I give them a DAP, they will accept it, and will surely go directly to buy an antibiotic. Because I don’t leave the prescriptions at the reception desk, I give them in person. And then you doubt, right? You don’t know if they’ve taken it or not, if you’re actually making a good….* (P7, DAP-Trial participant, medium-low socioeconomic area) *So, of course, if I don’t know how it is going to progress… without re-examining them, sometimes I’d rather be the one to decide when and how, than giving this to the patient.* (P25, DAP-Trial non-participant, medium-high socioeconomic area) *You have to think about it a lot and be really sure what you mustn’t give them [antibiotics], what you can give them, what you have to explain to them well… Putting time aside, it’s the act of thinking, it’s much easier to click, click, click, antibiotic, and goodbye. I mean, a DAP involves extra efforts from the clinician, apart from explaining and so on, even if you have a person on the other side of the desk who understands it perfectly, it implies thinking about it, saying, “Come on, let’s do it” and explaining it to them. I mean, I’m sure there are more DAPs at 3 pm than at 7 pm.* (P3, DAP-Trial participant, medium-high socioeconomic area)
	*Patient-led DAP versus DAP collection*	*The thing is, both as a professional and user of the system, I don’t think I would like the second option [DAP prescription collection] at all, because actually, if I’m fine, I won’t need it and I wouldn’t go get it; but if I’m ill and I really need to go get the antibiotic, it would mean the fever continues —it hasn’t decreased—, it would probably be a bacterial infection and I’m being forced to leave my house again or have to find someone to come with me . I mean, I find that when people are feeling bad, they are the ones to lose out in this case.* (P23, DAP-Trial non-participant, medium-high socioeconomic area) *If you leave it at the reception desk, the patient has to make an effort. Then, “this person may not come to collect it”, but they won’t schedule a visit either. This would be the ideal strategy, because they don’t come to the office, but they also don’t start taking it straight away, right?* (P14, DAP-Trial non-participant, medium-low socioeconomic area)
	*Improvement proposals*	*There could also be incentives for clinicians. Right? Make it a way to consider prescriptions, just as we have others, well, it could be one more.* (P4, DAP-Trial participant, medium-low socioeconomic area)
***DAP-Trial and primary care research barriers***	*DAP-Trial*	*(…) I found it very rewarding and interesting, partly because of what you see of an* *investment in the future, as promoting a rational use of drugs, of antibiotics, and for me it was very rewarding.* (P12, DAP-Trial participant, medium-low socioeconomic area) *Yes, because it’s useful. It’s a strategy… well, I didn’t know either, I learned to apply it as a result of the study we did last year. Did you apply it before?* (P10, DAP-Trial participant, medium-high socioeconomic area) *Well, for me the field work was very tedious. There were a lot of people who could have been included, but there was very little time, and that limited one a lot to include patients. On the other hand, I think I liked the study, because it was conducted in the primary care setting, in a real-world situation. Everything I was against in reporting the field work, I was in favour of after with the results, how they came out. But anyway, I really thought it was very tedious and that one lost interest in doing it because of what it meant if…* (P4, DAP-Trial participant, medium-low socioeconomic area)
	*Primary care research*	*“(…) that most of the research is conducted at the hospital level, but at the primary care level or with conditions that we only see in the primary care setting, such as upper RTIs, little has been done (…)* (P9, DAP-Trial participant, medium-high socioeconomic area) “*Hmmm, I think, firstly, that if you are working full-time as a healthcare provider, it is very complicated, because with our visiting hours, our timetable is almost full, and we don’t have much time left for anything else (…)* (P9, DAP-Trial participant, medium-high socioeconomic area) *It seems to me that perhaps now the only benefit —well, at least in the ICS [Catalan healthcare service]— that you can get is that, as an activity, it is valuable for your professional career. Well, I’m just saying this to try to see some personal benefit. The only thing I can think of right now. But apart from that…* (P2, DAP-Trial participant, medium-low socioeconomic area) I don’t know, if it is an interesting study for primary care and so interesting, then I think that the organization could collaborate with the schedules, or —I don’t know—, somehow saying, “if you do this, you will have fewer patients every day”, because it is harmful for patients who are waiting on you. Because you are feeling bad, thinking “now I will spend half an hour with this person, and I am already running 15 minutes late, or half an hour, add another half an hour and it will be an hour”, and the poor people there who had an appointment, you are also feeling bad about that, and so are they… it is also harming the healthcare service. I mean if the organization committed… (P9, DAP-Trial participant, medium-high socioeconomic area)

### Characteristics of RTI visits

#### The concept of RTI

Most uncomplicated RTIs were considered banal and self-limiting. Physicians commented that, with some patients, once informed that the infection was caused by a virus, they perceived this as the physician’s incapacity to determine the diagnosis or as not having any disease. RTIs were one of the main reasons for scheduled and unscheduled visits in winter, and patients tended to consult at very early RTI stages seeking a rapid cure. Some patients reconsulted every year and several times for each episode, and this despite previous experiences and having received appropriate information.

Despite RTIs being considered mostly banal, visits required a time investment in examining, informing, and educating patients, and in establishing a relationship of trust (if not previously established). However, this time investment was often not possible due to work loads and the structure of the healthcare system. RTI consultations were mainly motivated by patient self-perceptions of poor health, anxiety, and a fear of possible complications. These feelings varied depended on their own or acquaintances’ previous experiences and were often attributed to hearsay. According to some participants from centres with a medium-low socioeconomic level, poor health-related education was linked to a low socioeconomic status, while other participants considered that health knowledge among the general population had decreased from previous generations. It was considered that more education was needed, via primary care centres, the media, and schools.

Consultation often reflected the patient’s age, with young patients consulting because they were not used to being sick, and elderly patients consulting because they were concerned about their comorbidities. Another reason for consultation were requests for sick leave from work. Some participants were of the opinion that healthcare human resources were misused when patients consulted for mild cases of RTIs, with this misuse attributed to a lack of responsibility for self-care by patients. It was suggested that there was a need for patient empowerment, and also that access to rapid tests would be useful visit aid.

### Expectations and adequacy of antibiotic treatment

#### Physician-indicated treatment

Some patients expected a drug prescription to feel reassured or considered antibiotics to be an effective and fast-acting cure. Some patients felt that they were not being treated properly when recommended symptomatic medication (e.g., analgesics, antihistamines, mucolytics, and antitussives) while other patients even explicitly expressed dissatisfaction that antibiotics were not prescribed. There was a general opinion that the patient’s satisfaction was often greater when they were prescribed an antibiotic. Patients who accepted the non-prescription of antibiotics were those who had previously recovered without antibiotics, had experienced some adverse effects of antibiotics, or who were better informed about antibiotics, such as pregnant women.

### Inappropriate antibiotic use

Inappropriate use of antibiotics in the healthcare system was acknowledged. This inappropriate use in primary care was attributed mainly to defensive medicine based on low-cost drugs, most especially when there were time pressures or when there was no patient-physician relationship (e.g., unscheduled visits). Another reason was the presentation of some antibiotics does not fit with prescription patterns, meaning that patients typically have medication left over. Some participants suggested that this may be due to potential financial interests from pharmaceutical industry.

Despite recognizing the inappropriate use of antibiotics, there was a generalized opinion that primary care professionals make every effort to use them rationally, and even a trend in both hospital and primary care settings towards prescribing fewer antibiotics. This was attributed to better training and incentives for professionals, although such strategies were still considered to be insufficient. The trend to reduce antibiotic use was considered not to occur in the private health sector. In the opinion of the participants, inappropriate use of antibiotics was due to a range of opinions regarding both indication and choice of antibiotics. Private health sector physicians tended to prescribe more antibiotics and they are more often non-generic and expensive than public health sector physicians. Finally, it was acknowledged that doubts existed regarding the use of antibiotics because the criteria were always not clear.

### Patient self-medication

When patients visited, they had often already started symptomatic medication, and a typical recommendation was to follow the same treatment for a few more days. Occasionally, patients had already taken an antibiotic, typically left over from a previous prescription.

### DAP, how and for whom

#### Context

DAP was used in cases of doubts regarding aetiology, and was mainly used for pharyngitis in adults, and for acute middle-ear infection in paediatric patients. Taking into account information obtained in the patient-physician interaction, DAP was typically deployed in the following circumstances: before a weekend, travel, or an event; in unscheduled visits without follow-up; when enough time was available to appropriately inform the patient; and when the patient refused to leave without an antibiotic prescription even if not clinically indicated.

### Patient profiles

DAP may be indicated for specific patients, considering, most importantly, the patient’s capacity to understand the strategy. Candidates were also patients who were considered trustworthy, those with greater common sense (they probably would not use the antibiotics immediately), those with a relationship of trust with their physician, and those with chronic conditions who were knowledgeable about their pathology.

There was no consensus as to whether it was more difficult to implement DAP in young people who probably did not have a physician-patient relationship, or in older people with comorbidities or cognitive difficulties. It was agreed that DAP would not be indicated for patients experiencing anxiety, frequent healthcare users, or patients who insist on an antibiotic prescription.

### DAP advantages and disadvantages

Avoiding the need for a further visit was considered the main advantage of DAP, although the extent of the advantage was perceived to vary. A second advantage was that the DAP strategy generally satisfied both patient and physician. DAP also meant that patients had a safety net, in that they had the prescription if the condition deteriorated or failed to improve. DAP also represented an opportunity to educate patients that antibiotics are not always needed for RTIs and empowered them with greater decision-making autonomy. Finally, DAP as an alternative was useful when pursuing more rational use of antibiotics.

The main concern was uncertainty regarding patients’ proper use of the DAP strategy, mainly that they might use the antibiotic immediately. Some participants proposed that the prescription should not be available until a date recommended by physician. Related to this uncertainty, some physicians who did not use DAP stated that they preferred to take responsibility for the final clinical decision, despite the possibility of an additional visit. Two other physicians who did not use DAP considered that the patient had to be properly informed prior to being offered DAP and one physician considered that, with DAP, there was a possibility of antibiotics being prescribed despite not being indicated.

Some physicians who used DAP confirmed that it required a greater investment in time and effort, mainly in assessing whether the patient was a suitable candidate and then issuing instructions for use of the prescription. Possible professional responsibility in the event of a complication was expressed as a concern regarding the DAP strategy by one physician who used it.

### Patient-led DAP versus DAP collection

While DAP collection rather than patient-led DAP was considered by some to be a better strategy because immediate use was avoided, a recognized advantage of patient-led DAP was that it avoided a return visit by the patient.

### Improvement proposals

It was proposed that DAP use should be rewarded with incentives. It was also pointed out that deployment of DAP required more time and would need the health system’s educational role to be enhanced. Another proposal was to involve nurses and pharmacies in deployment of the DAP strategy.

### DAP-Trial and primary care research barriers

#### DAP-Trial

While some advantages to carrying out the DAP-Trial were commented, the main focus was on barriers. The main barrier perceived by both DAP-Trial participants and non-participants was the lack of time for the work implied by research. Perceived barriers by the DAP-Trial non-participants were the lack of suitable candidate patients and the disruption implied by DAP inclusion in routine practice. Perceived barriers by the DAP-Trial participants were the lack of support and a lack of agreement with recommendations to patients allocated to the DAP strategies. Some DAP-Trial participants found the study useful in making them more aware of and familiar with DAP, and interesting in that the study was implemented independently of the pharmaceutical industry. Also expressed was a feeling of belongingness, resulting from the follow-up emails periodically sent by the coordinating centre.

### Primary care research

It was recognized that research in the primary care compared to the hospital setting was scant, with a lack of time and poor rewards stated as the main barriers. Proposed in addition to involving nurses and residents in research, were incentives such as reducing work burdens and healthcare pressures, and the provision of financial rewards and additional holidays.

## Discussion

### Main findings

We identified a vicious circle between poor health-related education in patients with RTIs and time pressures in primary care centres. Time-consuming RTI consultations of poorly educated patients feeling anxious and fearful of possible complications, led to healthcare pressures that constrained physicians in terms of educating patients.

Physicians generally acknowledged inappropriate use of antibiotics in the health system, but also considered that they made every effort to prescribe them rationally, attributing inappropriate use to defensive medicine with low-cost drugs, based on a perceived trade-off between short-term negative consequences of non-prescription (i.e., complications) and long-term negative consequences of prescription (antimicrobial resistance).

DAP was therefore deployed in cases of doubt, in specific situations, and to specific patient profiles. The main advantage of DAP was considered to be the reduction in additional visits, while the main disadvantage was perceived to be uncertainty as to proper patient use. Regarding the DAP-Trial and primary care research, a lack of time was considered to be the main barrier to research in primary care settings. We did not find major differences between DAP-Trial participants and non-participants possibly because most of them used DAP in their practice.

#### Results in context

The decision to prescribe antibiotics for some RTIs depends not only on medical factors but also on patient-physician interaction factors [4, 13]. The results of our study corroborate previous studies in that the DAP strategy was considered useful for this kind of complex decision-making scenario [13, 18–23, 32].

Our study participants deployed DAP in cases of uncertainty and, as in previous studies, in specific situations, e.g., before the weekend or holidays [13, 19–21, 23, 32], as a negotiation strategy when patients insisted on antibiotics [13, 18–22, 32], and for certain patient profiles [13, 19–23, 32]. The main characteristics of DAP candidates that emerged in our study, consistent with previous studies, were patients capable of understanding the strategy [21, 23, 32], patients considered trustworthy [20, 32] and having common sense [32]. The patient-physician relationship was another key aspect to consider in deploying DAP, according to the results of our study. An issue that did not emerge in our study, unlike other studies, was that DAP was considered to strengthen this relationship [19–21, 23].

DAP was used by our study participants in apparently contradictory situations: (a) for patients who demanded antibiotics and refused to leave without a prescription, and for patients who were trusted not to immediately use the prescription (as in Hoye et al. [32] and Sargent et al. [21]); and (b) for patients consulting in unscheduled visits, in which the patient-physician relationship considered fundamental to this strategy was lacking. These apparently contradictory deployments of DAP, highlight the complexity of physician decision-making regarding antibiotic prescription.

DAP strategy advantages and disadvantages, in our study as in previous studies, are associated with the fact that DAP is a more patient-centred approach [18, 23]. Thus, while DAP provides the patient with a safety net [13, 18–22] since the prescription can be used if needed [32], and also empowers the patient by making them responsible for the final decision [18–21, 32], control is lost by the physician [13, 19, 20, 23].

Our study identified some important health system barriers to appropriate antibiotic use and DAP deployment, primarily the lack of a patient-physician relationship in unscheduled visits, poor patient health-related education, and the lack of professional time. These latter issues could be simultaneously addressed by nurses and pharmacists becoming more involved in educating patients regarding RTIs and their treatment. DAP was perceived, as in previous studies, as a golden opportunity for educating people about antibiotics [20, 21, 23, 32].

#### Limitations and strengths

The main limitation of our study is that the participants mostly came from primary care centres participating in the DAP-Trial, and most used DAP in their clinical practice. While the advantages of DAP may therefore be considered to be overestimated, our results are nonetheless consistent with the extant literature. A second limitation is that our study did not include participants from rural settings, although Fletcher et al. [22] found no differences between rural-urban contexts in their study. A third limitation is that, due to the few professionals participating in the DAP-Trial, two FGDs were not homogeneous in terms of the socioeconomic level of the centre’s population. Furthermore, a nurse who participated in the DAP-Trial was included in a FGD. Including this participant granted the feasibility of one of the groups. The researchers involved in conducting and analysing the FGD assessed that the dynamics were not negatively affected, and, indeed, the nurse’s contributions were particularly enriching.

A major strength of our study is that it included professionals who had deployed DAP and so were well aware of the positive and negative aspects of DAP. A second strength is that, as far as we are aware, this is the first qualitative study of professionals and DAP conducted in a country in southern Europe, where antibiotic use is comparatively higher than in northern Europe [6]. Finally, our study complements several other studies published by our group [11, 17, 24, 33] aimed at raising awareness and improving implementation of the DAP strategy.

#### Implications for practice and research

Our findings highlight the fact that time pressures, poor health-related education of patients, and the lack of a patient-physician relationship in unscheduled visits were important barriers to optimal antibiotic use and to deployment of the DAP strategy in primary care. Policy-makers may therefore consider strategies, such as the following to overcome these challenges: (i) the provision of RTI health-related education and self-care, and the encouragement of proper use of healthcare services supported by primary care nurses and pharmacists; (ii) improved access to rapid streptococcal testing; and (iii) reorganization of physician agendas so that RTI consultations are attended by the referring physician whenever possible.

Another implication of our findings is that they point to a lack of consensus about some of the criteria to be considered by physicians in deploying the DAP strategy. This suggests that clinical guidelines on RTI management in primary care need to better specify criteria for deployment of DAP, including patient and contextual factors which should be considered when using DAP strategies, as well as the standardization of prescription use recommendations for patients. Finally, the poor health-related education of patients was one of the main themes that emerged in this study. A recent systematic review showed that educational interventions were one of the most efficacious and safe strategies for optimal antibiotic prescribing for RTIs [16]. Given the need for further studies to evaluate RTI educational interventions for patients, our group is conducting a multicentre factorial trial of two educational interventions, targeting both parents and professionals.

## Conclusions

DAP was used by participants in cases of doubt, in specific situations, and for specific patient profiles. Weak points were detected in our primary care system and in its users that affect the proper use of both antibiotics and DAP, namely, time pressures on professionals, poor patient health-related education, and the lack of a patient-physician relationship in certain scenarios. Proposed to overcome these challenges are educational interventions regarding RTIs and optimal use of healthcare resources and the formulation of better DAP-related recommendations in guidelines.

### Electronic supplementary material

Below is the link to the electronic supplementary material.

Supplementary Material 1

## Data Availability

The data will be made available to researchers who provide a methodologically sound proposal for use. Proposals should be submitted to the corresponding author.

## List of Abbreviations

DAP: Delayed antibiotic prescription
FGD: Focus group discussion
IAP: Immediate antibiotic prescription
NAP: No antibiotic prescription
RTI: Respiratory tract infection
