# Supplementary material for: Perceptions and attitudes regarding delayed antibiotic prescription for respiratory tract infections: a qualitative study
Source: BMC Prim Care. 2023 Oct 4;24:204. doi: 10.1186/s12875-023-02123-4 (PMC10548630; doi:10.1186/s12875-023-02123-4)
Supplement: Supplementary file 1 — Supplementary Material 1 [file 12875_2023_2123_MOESM1_ESM.docx]

**Additional file 1**

**Appendix 1. Script for focus group discussions**

**Theme 1. Acute uncomplicated respiratory tract infections (RTIs)**

-What is your opinion of acute upper RTIs and why?

-What is your opinion regarding the seriousness of RTIs and why?

-How do you usually deal with RTIs? Why?

-How long do you think it takes for RTIs to resolve? Why?

**Theme 2. Medical consultations for acute uncomplicated RTIs and recommendations**

-In relation to your care routines, what do you usually recommend to patients who consult for RTIs and why?

-What expectations do you think patients have about non-antibiotic and antibiotic medications? Why?

-What kind of treatment do you think patients expect and why?

**Theme 3. Antibiotic medication in acute uncomplicated RTIs**

-What is your opinion of antibiotics as a treatment for acute RTIs and why?

- Do you think antibiotics are prescribed properly and why?

**Theme 4. Delayed antibiotic prescription (DAP) strategy in acute uncomplicated RTIs**

-What is your opinion of using a DAP strategy in patients with upper RTIs and why?

-What do you think are the advantages and disadvantages of DAP and why?

For those who use the DAP strategy in their clinical practice:

- What do you expect when you use the DAP strategy? In what situations do you use it? What kind of patient is a candidate? Why?

-How do you perceive the reaction of patients when you prescribe DAP? Why?

-How do you feel about this type of treatment strategy and why?

-When in doubt, what factors lead you to prescribe antibiotic to be started immediately or to be delayed and why?

-What is your opinion of the two types of DAP strategies: (1) a delayed patient-led strategy in which the patients receives the prescription, with instructions to only use it if the RTI worsens or fails to improve, and (2) a delayed collection strategy in which the patient collects their prescription from the primary care centre after 72h if they think they need it.

For those who participated in the DAP trial:

**Theme 5. Participation in the DAP trial**

Describe your experience with the DAP trial...

- What did you think of the experience and why?

- What did you assess of it and why?

**Appendix 2. Script for individual semi-structured interviews**

**Theme 1. Medical consultations for acute uncomplicated RTIs**

-Why do patients consult for RTIs? Why do patients seek antibiotic treatment? Why do patients seek symptomatic treatment? Due to sick leave? For immediate symptom relief?

**Theme 2. Antibiotic use for acute uncomplicated RTIs**

-Regarding clinical guidelines on RTIs in primary care: are they lacking, not disseminated, or not known among healthcare professionals?

**Theme 3. Use of DAP for acute uncomplicated RTIs**

-What is your opinion on the use and implementation of the DAP?

-Do you think that DAP is a good general strategy or for specific cases?

-Do you use the DAP strategy sporadically, only if you have the "right patient" to prescribe it to, or if there is enough time for the consultation? Why?

- How would you define the "right patient" and why?

-Why is the DAP strategy considered to require more consultation time?

-Some physicians are concerned about the lack of awareness of episode outcomes when DAP is used: why this concern?
